# Differences in the microenvironment for leisure physical activity and commuting in São Paulo

**DOI:** 10.11606/s1518-8787.2026060006996

**Published:** 2026-06-12

**Authors:** Elaynne Silva de Oliveira, Inaian Pignatti Teixeira, Ítalo Vinícius Floriano de Paula, Jader Correia de Lacerda, Rildo de Souza Wanderley, Alexandre Augusto de Paula da Silva, Adriano Akira Ferreira Hino, Alex Antonio Florindo

**Affiliations:** I Universidade Federal do Maranhão. Pinheiro, MA, Brasil; II Universidade do Estado de Minas Gerais. Passos, MG, Brasil; III Universidade de São Paulo. Faculdade de Medicina. São Paulo, SP, Brasil; IV Universidade de São Paulo. Escola de Artes, Ciências e Humanidades. São Paulo, SP, Brasil; V Universidade de Pernambuco. Escola Superior de Educação Física. Recife, PE, Brasil; VI Washington University in St Louis. School of Public Health. Prevention Research Center. St. Louis, MO, United States; VII Pontifícia Universidade Católica do Paraná. Escola de Ciências da Vida. Programa de Pós-Graduação em Ciências da Saúde. Curitiba, PR, Brasil

**Keywords:** Built Environment, Physical Activity, Inequities

## Abstract

**OBJECTIVE:**

To describe the characteristics of the built environment at the micro-scale and to examine differences according to residential area and social and demographic variables among adults in São Paulo, Brazil.

**METHODS:**

This is a cross-sectional study involving 1,434 adults from the ISA–Physical Activity and Environment cohort. Environmental characteristics were assessed using the Microscale Audit of Pedestrian Streetscapes (MAPS Global) method based on Google Earth images along 400–700-meter routes centered on the participants’ georeferenced addresses. For the analysis, Mann-Whitney U tests were used for two independent groups and Kruskal-Wallis tests for three or more groups, followed by Dunn’s post-hoc test, to assess differences in mean scores by health coordination unit, sex, age, educational level, and skin color.

**RESULTS:**

The surroundings of residences in the Central-West region had the highest overall scores (p < 0.001). Individuals with higher education levels, who self-identified as White or Asian, and those aged 60 years or older resided in areas with better scores for aesthetics, social aspects, presence of trees, canopy cover, sidewalks, intersections, and overall environmental score (p < 0.001). In contrast, people with lower educational attainment and those who self-identified as Black or Brown lived in areas with poorer sidewalk conditions (p < 0.001).

**CONCLUSION:**

It is concluded that micro-scale conditions were better in the vicinity of residences in central regions and for individuals with higher education, White or Asian skin color, and older age, highlighting the need for public policies aimed at more equitable and inclusive urban planning.

## INTRODUCTION

The urban environment and city planning influence the health of the population, affecting factors such as sedentary lifestyles, low levels of physical activity, air and noise pollution, violence, and social isolation^1^. In countries with historically limited access to adequate urban infrastructure, these relationships tend to be more evident, especially given rapid population growth in urban centers and urbanization processes marked by decades of insufficient planning^2^. In the Brazilian context, a country classified as upper-middle-income country by the World Bank, it is estimated that, in 2023, approximately 85% of the population lived in urban areas^3^, and it is projected that, by 2050, approximately 70% of the world’s population will reside in these areas⁴. This rapid urbanization poses a global challenge, as cities become central settings for the production of health inequities⁵, reinforcing the importance of urban planning aimed at promoting healthier cities.

Studies on physical activity highlight health disparities, with evidence that gender^6^, income^7^, education level^8^, and race/ethnicity^9^ influence these practices, particularly in leisure and as a means of transportation. Although individual factors are relevant, interaction with the environment is crucial. Neighborhoods, housing, and communities impact health^10^, underscoring the importance of urban planning for public health. Evidence from high-income countries such as the United States, England, and Australia, according to the World Bank’s classification^11^, indicates that urban space tends to be unequal for more vulnerable groups^12^. Despite socioeconomic and cultural differences, in Brazil, a study in Curitiba showed a lower presence of trees and aesthetic elements in the outskirts, reflecting similar inequalities^13^.

Accessible public spaces, high-quality sidewalks, road connectivity, and traffic safety are essential for promoting physical activity, especially in low- and middle-income countries^14^. São Paulo has been characterized by haphazard urban planning, driven by rapid industrialization and unregulated migration and immigration processes. For example, between 1950 and the 2000s, the population rose from two million to over 10 million inhabitants^15^. The lack of planning led to problems such as spatial inequality, access to public transportation, urban mobility, basic sanitation, soil sealing, air and noise pollution, flooding, inequities in green spaces, housing shortages, and substandard housing.

However, starting in 2010, important policies were implemented to reduce environmental inequities^16^. Between 2015 and 2020, there was an increase in infrastructure for physical activity, such as outdoor gyms, squares, parks, and bike paths, driven mainly by the New Master Plan and the Urban Mobility Plan. However, these improvements are still more common in areas with higher socioeconomic status. For example, Teixeira et al.^17^ demonstrated that, despite the growth of structures that encourage physical activity, such as bike lanes, these facilities are still predominantly found in areas with higher socioeconomic status within the city. Microenvironmental or microscale variables, such as the presence and quality of sidewalks, traffic controls like traffic lights and speed bumps, aesthetic aspects, and green cover such as the presence of trees, are very important for promoting physical activity.

However, there remains a significant gap in the literature regarding research on the microenvironment for physical activity, especially in low- and middle-income countries like Brazil, and in cities marked by deep inequalities, such as São Paulo. The capital of São Paulo faces complex problems such as high population density, chaotic traffic, and income inequality, which can impact public health. Therefore, it is important to conduct an in-depth investigation of the microenvironment for physical activity practices, both for leisure and as a means of transportation, in cities like São Paulo, Brazil, which well represent the characteristics of Global South megacities, where many social and environmental inequalities still exist. This study may inform public policies on housing, urban infrastructure, urban mobility, health surveillance, and the promotion of healthy environments. In light of the above, the present study aims to describe the micro-scale characteristics of the environment for physical activity and to examine possible differences according to residential areas, educational levels, and skin color among adults in the city of São Paulo, Brazil.

## METHODS

### Research Setting

São Paulo is the most populous city in Brazil and Latin America, with an estimated population of 11,451,999 inhabitants, covering an area of 1,521.110 km^2^ and a population density of 7,528.26 inhabitants per km^2^. The city is divided into 96 districts, 31 sub-districts, six health zones, and in 2022 had 27,301 census tracts. São Paulo accounts for approximately 11% of the country’s gross domestic product and has a Gini index of 0.539, which ranks it among the cities with the highest income inequality, a factor that may impact access to healthcare, housing, and transportation^3,18^.

### Type of Study

This is a cross-sectional study that uses data from the “*Inquérito de Saúde de São Paulo* (ISA): *Atividade Física e Ambiente* (São Paulo Health Survey [ISA]: Physical Activity and Environment)” cohort. Its primary objective is to investigate whether the built environment is associated with physical activity during leisure time and as a means of transportation, in addition to secondary objectives such as the study of sedentary behavior, obesity, cardiovascular diseases, and mental health conditions among adults residing in the city of São Paulo. Further details on the ISA–Physical Activity and Environment can be found in the study by Florindo et al.^19^

### Sample

The sample for this study included 1,434 individuals interviewed during the second wave (2020/2021), who reside in the six administrative health regions (East, Southeast, Central-West, North, and South) of the city of São Paulo. Further details on the ISA–Physical Activity and Environment sampling can be found in an article published by Florindo et al.^20^ that describes the cohort profile.

### Questionnaires

The ISA–Physical Activity and Environment study used a structured questionnaire administered via telephone interviews in the second wave (2020/2021) due to the Covid-19 pandemic. Data collection was conducted using the Computer Assisted Telephone Interviewing methodology, similar to that employed in the Ministry of Health’s *Sistema de Vigilância de Fatores de Risco e Proteção para Doenças Crônicas por Inquérito Telefônico* (Surveillance System for Risk and Protective Factors for Chronic Diseases via Telephone Survey).

The second-wave questionnaire consisted of 70 questions, organized into 11 thematic blocks: personal identification (six questions), physical activity (30 questions), mobility (four questions), sleep assessment (three questions), weight and height (two questions), health perception (one question), diseases (eight questions), smoking (one question), behavior during the pandemic (three questions), socioeconomic characteristics (two questions), and family and household characteristics (10 questions).

### Assessment of the Microenvironment for Physical Activity

The microenvironment for physical activity, also understood as the microscale level of assessment, refers to the small-scale environment surrounding individuals, whose conditions and characteristics can significantly influence the decision to engage in physical activity during leisure time or as a means of transportation^10^. In general, the microenvironment involves assessments of streets, segments, and intersections and examines the presence and quality of sidewalks, the presence and quality of spaces such as bike lanes, parks, and squares, traffic safety, assessing the presence of traffic controllers, aesthetic aspects such as landscaping and graffiti, the presence of lighting structures, vegetation cover, the presence and size of buildings, and the presence of commercial services, educational and religious institutions, and public and private facilities.

The microenvironment assessment was conducted using the Microscale Audit of Pedestrian Streetscapes-Global (MAPS-Global)^21^. A total of 1,434 adults assessed in the second wave of the study (2020/2021) were included, and their 1,205 residential addresses were georeferenced to construct the routes. The audit was conducted online using Google Earth imagery along routes of 400 and 725 meters, a distance corresponding approximately to the distance a person can walk in up to ten minutes. This range is in accordance with the standard adopted by the MAPS Global instrument manual. Google Street View was used to traverse these routes. Based on the addresses, the commercial center was identified as the census tract with the highest concentration of records in the National Registry of Legal Entities (CNPJ), obtained via the São Paulo Urban Property Tax. The 794,968 CNPJ records were georeferenced, and each census tract received a spatial marker indicating its commercial center.

To increase the coverage of the assessment, we chose to analyze both sides of the streets (right and left). The environmental assessment took place between December 2020 and September 2022. In total, 1,205 routes, 11,276 segments, and 11,222 intersections were analyzed, with an average time of 25.8 minutes per route (SD = 17.4 minutes). The images used in Google Street View ranged from January 2010 (oldest) to May 2022 (most recent).

The audit was conducted by a team of seven pre-trained assessors, three of whom also served as supervisors during the data collection process. The supervisors received specific training provided by the MAPS coordinating group in Brazil and were subsequently responsible for training the other assessors involved in the study. As part of the standardization and quality control process, all auditors underwent inter-rater reliability tests, as recommended by the MAPS method. Only evaluators who achieved the minimum level of agreement of 90% required by the instrument participated in data collection. All methodological procedures are described in detail in the study by Oliveira et al.^22^

The items assessed by MAPS Global use different response formats (dichotomous, categorical, and continuous), except for the land-use questions, which range from 0 to 5. To calculate scores, dichotomous responses (no/yes) are scored as 0 or 1. Individual items and subscales are scored, resulting in subscales of positive valences (associated with a greater likelihood of engaging in physical activity) and negative valences (associated with a lower likelihood of engaging in physical activity). The scales and subscales generate the scores for each section (routes, segments, and intersections). For analyses and calculations, the standardization and systematization of scores adopted by the instrument’s methodology were followed.

### Study Variables

#### Microenvironment variables

To generate the built environment scores, three sections of the instrument were considered: routes, segments, and intersections, totaling 148 items organized into scales and main scores of the MAPS Global*.* The routes section analyzes the diversity of urban infrastructure, including residential and commercial land use, availability of services, mobility, and the aesthetic quality of the environment. The segments section examines in detail the stretches between intersections, considering infrastructure for pedestrians and cyclists, sidewalk characteristics, the presence of shade, street vendors, and elements that impact pedestrian safety and experience. The intersections section, meanwhile, assesses accessibility and safety for crossing, examining signage, road width, traffic lights, ramps, and crosswalks.

For the analysis, the following scales were considered: mixed land use, street use, aesthetics and social environment, pedestrian infrastructure, trees and canopy cover, sidewalks, negative sidewalk characteristics, pedestrian amenities, and accessibility. The main scores, routes, segments, intersections, and the overall score of the instrument were also analyzed. The segments and intersections were presented as averages per route, avoiding individual discrepancies. Initially, the variables of these elements were summed, and then the averages of the attributes were calculated, before summing the overall scores of each subscale, scale, and overall score. In the analysis of the routes, it was not necessary to calculate averages, as each individual had only one route.

## Demographic, Social, and Residential Region Variables

Data were included for individuals aged 18 years or older (n = 1,434, residing in 1,205 households) assessed up to the second wave of the study (2020/2021). The variables considered were: sex (men and women) and skin color (White and Asian; Black, Brown, and Indigenous), both collected at baseline and used as a reference throughout the cohort; educational attainment (level 1: up to the 5th grade of elementary school; level 2: up to the 2nd year of high school; level 3: complete high school; and level 4: incomplete higher education or higher), and age (18 to 39 years; 40 to 59 years; and 60 years or older), both collected in the second wave.

The participants’ regions of residence, as classified by the Health Coordinating Offices in effect at the study’s baseline in 2014/2015 (North, South, East, Southeast, and Central-West), were used due to the sample selection and for potential comparisons of the microenvironment surrounding the households where people lived.

## Data Analysis

Kolmogorov-Smirnov tests were conducted to determine whether the variables followed a normal distribution, revealing that all variables related to the built environment had a non-parametric distribution. For the descriptive analyses, medians, interquartile ranges, minimum and maximum values were calculated, as well as the percentage of missing values for the attributes aggregated by scales and in the sections on routes, segments, and intersections.

Differences in mean scores for the built environment were analyzed based on different residential regions, educational levels, skin color, and age groups. Subsequently, Mann-Whitney U tests were performed for two independent groups and Kruskal-Wallis tests for three or more groups, followed by Dunn’s post-hoc test to identify statistically significant groups.

All data were analyzed using SPSS version 24.0 (SPSS, Inc., Chicago, IL). For all tests, statistical significance was set at p < 0.05.

## Ethical Issues

The study was approved by the Ethics Committees of the Escola de Artes, Ciências e Humanidades da Universidade de São Paulo (10396919.0.0000.5390) and the Municipal Health Secretariat of São Paulo (10396919.0.3001).

## RESULTS

Most of the sample was female, aged up to 59 years, with a high school or higher education level, self-identified as white or asian, and residing in the southern and southeastern health districts (Table 1).

**Table 1:** Social and demographic characteristics of the sample in 2020/2021, ISA-Physical Activity and Environment study, São Paulo, SP, Brazil.

Sociodemographic variable	n	%
Gender		
Female	840	58.6
Male	594	41.4
Age groups		
18 to 39 years old	489	34.1
40 to 59 years old	432	30.1
60 years and older	513	35.8
Education level^a^		
Up to 5th grade of elementary school	251	17.5
Up to 10th grade	284	19.8
Complete high school	470	32.8
Incomplete higher education or higher	419	29.2
Skin color^a^		
White and Asian	768	53.6
VBlack, Brown, and Indigenous	656	45.7
Health and Housing Coordination Offices		
Central-West	201	14.0
East	259	18.1
North	279	19.5
Southeast	326	22.7
South	369	25.7
Total	1,434	100

ISA: São Paulo Health Survey.

^a^Ten people with missing values.

It was found that attributes such as mixed land use, trees, canopy cover, and sidewalks are present on most routes. However, features such as infrastructure and pedestrian accessibility are lacking on half or more of the routes (Table 2).

**Table 2:** Scales and overall scores of the routes assessed by MAPS Global according to participants’ residential addresses (n = 1,434 of 1,205 addresses assessed), ISA-Physical Activity and Environment study, São Paulo, SP, Brazil.

MAPS Scale and Score	Items/points	Median	Interquartile range	Minimum–Maximum	% missing
Scale					
Mixed land use	38 (0 to 162)	17.0	12 to 21	0 to 43	0.1
Street use	20 (0 to 24)	5.0	2.7 to 7.0	0 to 15	5.3
Aesthetics and social environment	5 (0 to 11)	2.0	1.0 to 4.0	-3 to 9	19.7
Pedestrian infrastructure	3 (0 to 3)	0.1	0 to 0.3	0 to 1	88.0
Trees and canopy cover	3 (0 to 21)	3.5	2.6 to 4.6	0 to 10	0.9
Sidewalks	2 (0 to 12)	9.2	8.2 to 10.3	0 to 12	0.1
Negative characteristics on sidewalks	6 (0 to 36)	10.5	8.8 to 12.7	0 to 20	0.1
Pedestrian facilities	7 (0 to 7)	0.5	0.2 to 0.7	0 to 3	49.3
Accessibility	3 (0 to 6)	0.0	0 to 0.5	0 to 6	69.7
Score					
Routes		24.0	18 to 30	0 to 57	
Segments		10.8	8.5 to 14.2	-4 to 39	
Intersections		1.0	0.3 to 2.0	-1 to 13	
Overall instrument score		37.1	28.7 to 45.9	1 to 105	

ISA: São Paulo Health Survey; MAPS: Microscale Audit of Pedestrian Streetscapes.

The surroundings of the residences of people living in the Central-West region obtained the highest scores on the scales of aesthetics, trees and canopy cover, accessibility, routes, segments, intersections, and overall environment score when compared to the surroundings of the other regions. The Central-West region also had the lowest score for negative sidewalk characteristics. In contrast, the South region had lower scores for aesthetics and pedestrian-friendliness (Table 3).

**Table 3:** Scales and overall microenvironment scores assessed in the vicinity of individuals’ residences (n = 1,434 out of 1,205 addresses) according to residential regions established by health coordination offices in 2015, ISA-Physical Activity and Environment Study, São Paulo, SP, Brazil.

	MAPS scale and score				
	Central-West (1)	East (2)	North (3)	Southeast (4)	South (5)
	**Median (interquartile range)**				
Scale					
Mixed land use	16.0 (9.0–24.0)	18.0 (14.0–21.0)	17.0 (12.0–21.0)	14.0 (10.0–18.25)^a^	18.0 (12.0–23.0)
Street use	5.0 (3.0–7.0)	5.0 (2.0–7.0)	5.0 (2.75–7.0)	5.0 (2.0–7.0)	5.0 (2.0–7.0)
Aesthetics and social environment	3.0 (2.0–5.0)^a^	2.0 (1.0–3.0)	2.0 (1.0–4.0)	3.0 (2.0–4.0)	1.0 (0–3.0)^a^
Pedestrian infrastructure	0.1 (0–0.3)^a^	0 (0–0.2)	0.1 (0–0.3)	0 (0–0.2)^a^	0.1 (0–0.3)
Trees and vegetation cover	4.7 (3.3–6.7)^a^	3.25 (2.4–4.2)	3.5 (2.6–4.6)	3.6 (2.9–4.6)	3.1 (2.5–4.0)
Sidewalks	9.3 (8.1–10.4)	9.7 (8.4–10.7)	9.2 (8.2–10.3)	9.1 (8.1–10.0)	8.8 (8.0–9.8)^a^
Negative characteristics of sidewalks	9.8 (8.4–11.1)^a^	11.2 (8.7–13.0)	10.5 (8.4–13.0)	10.8 (8.5–12.0)	11.1 (9.7–13.1)
Pedestrian facilities	0.5 (0.2–1.0)	0.4 (0.2–0.7)	0.5 (0.2–0.7)	0.5 (0.25–0.78)	0.3 (0–0.6)^a^
Accessibility	0.2 (0.0–1.9)^a^	0 (0–0.5)	0 (0–0.5)	0 (0–0.5)	0 (0–0.5)
Score					
Routes	24.0 (15.0–34.0)^a^	23.0 (19.0–31.0)	24.00 (18.0–30.0)	22.0 (17.0–26.0)	24.0 (19.0–31.0)
Segments	16.4 (10.5–21.8)^a^	10.5 (8.8–13.4)	10.8 (8.5–10.8)	11.5 (7.2–14.7)	9.4 (6.2–11.2)
Crossings	1.3 (0.5–3.7)^a^	0.7 (0.2–1.8)	1.0 (1.0–2.0)	1.1 (0.5–2.0)	0.8 (0–1.5)
Overall instrument score	43.0 (32.5–57.3)^a^	36.0 (28.6–46.5)	37.1 (28.7–45.9)	35.42 (27.2–44.8)	34.5 (26.7–43.3)

ISA: São Paulo Health Survey; MAPS: Microscale Audit of Pedestrian Streetscapes.

Note: p < 0.05 = Kruskal-Wallis test.

^a^ Statistically significant value and different from the other medians.

When comparing the surroundings of residences according to people’s educational levels (Table 4), individuals with a college degree had higher scores on the positive scales of aesthetics and social environment, trees and canopy cover, pedestrian-friendliness, accessibility, route scores, segments, intersections, and overall environment score, as well as lower scores for negative sidewalk characteristics.

**Table 4:** Scales and overall scores for the microenvironment surrounding residences according to the sample’s educational levels (n = 1,424 out of 1,205 addresses), ISA–Physical Activity and Environment study, São Paulo, SP, Brazil.

MAPS scale and score	Education Level			
	Level 1	Level 2	Level 3	Level 4
**Median (interquartile range)**				
Scale				
Mixed land use	17.0 (11.0–21.0)	17.0 (12.0–21.0)	17.0 (12.0–21.0)	16.0 (11.0–21.0)
Street use	5.0 (2.0–7.0)	5.0 (2.0–7.0)	5.0 (2.0–7.0)	5.0 (3.0–8.0)
Aesthetics and social environment	2.0 (1.0–3.0)	2.0 (1.0–3.0)	2.0 (1.0–4.0)	3.0 (1.0–4.0)^a^
Pedestrian infrastructure	0.1 (0–0.3)	0.1 (0–0.3)	0.1 (0–0.2)	0.1 (0–0.3)
Trees and canopy cover	3.3 (2.5–4.4)	3.2 (2.4–4.2)	3.4 (2.6–4.5)	3.8 (3.0–5.4)^a^
Sidewalks	9.2 (8.1–10.4)	9.1 (8.0–10.1)	9.3 (8.2–10.3)	9.3 (8.3–10.3)
Negative characteristics of sidewalks	11.1 (9.1–13)	10.5 (9.0–12.7)	10.6 (9.0–13.0)	10.2 (8.6–12.0)^a^
Pedestrian-friendly features	0.4 (0.2–0.7)	0.4 (0.1–0.6)	0.5 (0.2–0.8)	0.5 (0.2–0.9)^a^
Accessibility	0 (0–0.5)	0 (0–0.5)	0 (0–0.5)	0 (0–0.9)^a^
Score				
Routes	24.0 (17.0–31.0)	24.0 (19.0–30.0)	23.0 (18.0–29.0)	24.0 (18.0–30.0)
Segments	10.1 (7.8–13.2)	10.5 (8.5–13.4)	10.6 (8.2–13.9)	12.1 (9.0–17.7)^a^
Crossings	1.0 (0.3–1.7)	0.8 (0.2–1.6)	1.0 (0.4–2.0)	1.1 (0.4–2.7)^a^
Overall instrument score	35.8 (26.8–45.7)	35.7 (28.4–45.5)	36.6 (28.6–44.7)	39.2 (31.3–49.0)^a^

ISA: São Paulo Health Survey; MAPS: Microscale Audit of Pedestrian Streetscapes.

Note: p < 0.05 = Kruskal–Wallis test.

^a^ Statistically significant value and different from the other medians.

It was found that people aged 60 years or older had the highest scores on the positive scales for aesthetics, presence of trees, and canopy cover, as well as the segment score. Furthermore, this group had lower scores on negative sidewalk characteristics (Table 5).

**Table 5:** Scales and overall microenvironment scores according to age groups and skin color (n = 1,434 out of 1,205 addresses), ISA-Physical Activity and Environment study, São Paulo, SP, Brazil.

MAPS scale and score	Age group (age)		
	18 to 39 years	40 to 59 years	≥ 60 years
**Median (interquartile range)**			
Scale			
Mixed land use	17.0 (12.0–21.0)	16.0 (12.0–21.0)	17.0 (11.0–21.0)
Street use	5.0 (2.0–7.0)	5.0 (3.0–7.0)	5.0 (3.0–8.0)
Aesthetics and social characteristics	2.0 (1.0–4.0)	2.0 (1.0–4.0)	**3.0 (1.0–4.0)** ^a^
Pedestrian infrastructure	0.1 (0–0.3)	0.1 (0–0.3)	0.1 (0–0.2)
Trees and canopy cover	3.5 (2.7–4.4)	3.3 (2.6–4.5)	**3.6 (2.7–4.8)** ^a^
Sidewalks	9.3 (8.2–10.3)	9.2 (8.1–10.2)	9.2 (8.2–10.3)
Negative characteristics of sidewalks	10.6 (9.0–12.8)	10.6 (9.0–13)	**10.2 (8.7–12.3)** ^a^
Pedestrian amenities	0.5 (0.1–0.7)	0.4 (0.2–0.7)	0.5 (0.2–0.7)
Accessibility	0 (0–0.5)	0 (0–0.5)	0.1 (0–0.6)
Score			
Routes	24.0 (18–30.0)	23.0 (18–29)	24.0 (17–30)
Segments	10.5 (8.0–13.7)	10.6 (8.5–13.8)	**11.5 (8.6–15.3)** ^a^
Crossings	0.9 (0.3–2.0)	0.9 (0.3–2.0)	1.1 (0.4–2.0)
Overall instrument score	37.0 (28.5–45.6)	36.4 (28.6–45.7)	37.7 (28.7–46.9)
**MAPS scale and score**	**Skin color**		
	**Whites and Asians**	**Blacks and browns**	
**Median (interquartile range)**			
Scale			
Mixed land use	18.0 (12.0–24.0)	**19.0 (14–24.0)**	
Street use	5.0 (2.0–8.0)	5.0 (2.0–7.0)	
Aesthetics and social characteristics	**2.0 (1.0–4.0)** ^a^	2.0 (0–3.0)	
Pedestrian infrastructure	**0.1 (0–0.3)**	0.1 (0–0.2)	
Trees and canopy cover	**3.6 (2.8–4.7)** ^a^	3.2 (2.5–4.2)	
Sidewalks	**9.4 (8.3–10.3)**	9.1 (8.0–10.1)	
Negative characteristics of sidewalks	10.2 (8.6–12.4)	**10.7 (9.1–13.0)** ^a^	
Pedestrian amenities	**0.5 (0.2–0.8)**	0.4 (0.1–0.7)	
Accessibility	**0.1 (0–0.7)**	0 (0–0.5)	
Score			
Routes	24.0 (18.0–30.0)	24.0 (17.7–29.0)	
Segments	**11.5 (8.8–15.1)** ^a^	10.0 (7.9–13.6)	
Crossings	**1.1 (0.4–2.3)** ^a^	0.8 (0.2–1.7)	
Overall instrument score	**38.8 (29.7–47.0)** ^a^	35.4 (27.2–43.8)	

ISA: São Paulo Health Survey; MAPS: Microscale Audit of Pedestrian Streetscapes.

Note: p < 0.05 = Kruskal-Wallis test for age and Mann–Whitney U test for skin color.

^a^ Statistically significant value; medians in bold are statistically different from those not in bold, indicating significant differences between the compared groups.

Regarding skin color, self-identified White and Asian individuals had higher scores for pedestrian infrastructure, accessibility, aesthetics, presence of trees and canopy cover, segments, intersections, and the overall environmental score. On the other hand, the highest scores for mixed land use and negative sidewalk characteristics were observed among self-identified black and brown individuals (Table 5).

## DISCUSSION

This study aimed to describe the characteristics of the built microenvironment among adults and older adults participating in the ISA-Physical Activity and Environment cohort and to examine differences according to regions of residence and demographic and social characteristics. Individuals residing in central regions, with higher educational attainment, and of White or Asian skin color had higher scores for environments conducive to recreational physical activity and commuting.

Regarding results by region of residence, it is known that in São Paulo, SP, central areas have a higher concentration of people with higher socioeconomic status. Furthermore, studies demonstrate that structures that favor the practice of leisure physical activity and active commuting, such as train and subway stations, bike paths, and public squares, are more prevalent in these central regions of the city⁸. These findings highlight the existence of territorial inequalities in the distribution of built environment attributes associated with physical activity.

Although still scarce, studies evaluating the microenvironment for physical activity in Brazil and Latin American countries, especially those using standardized environmental audit tools such as MAPS, indicate a consistent pattern of spatial inequality. A study conducted in Curitiba^13^, Brazil, using MAPS, reinforces this scenario by assessing the microenvironment around 30 public schools; it demonstrated that the quality of the built environment decreases as one moves away from the urban center, with higher scores in central areas and poorer conditions in peripheral regions. Furthermore, the study also showed that these peripheral areas, predominantly inhabited by lower-income groups, have less access to urban services and aesthetic and comfort-enhancing elements, such as tree-lined streets and urban art.

Meanwhile, a study conducted in the municipality of Cambé^23^, in the state of Paraná, used cluster analysis to examine groupings formed based on the relationship between micro-scale walkability, income by census tract, and walking levels—the latter obtained from the Cambé (PR) Mobility Plan. It showed that regions with higher income and those with greater pedestrian traffic had the highest average overall walkability scores in their respective sections. These findings reinforce the existence of a positive association between better conditions of the built environment at the micro-scale and the practice of walking, while also revealing socio-spatial inequalities in the distribution of urban infrastructure favorable to active mobility.

Discrepancies in the quality of the urban environment in Brazil result from historical, legal, and social inequality factors, reflecting processes of disorderly urbanization and irregular occupation, as observed in São Paulo, where significant contrasts in infrastructure and living conditions persist^24^. Data from the 2022 Demographic Census^25^ reinforce that these inequalities are strongly associated with residential locations, showing that residents of favelas and urban communities live with significantly more precarious infrastructure, especially regarding key attributes of the pedestrian micro-scale, such as sidewalks, accessibility, tree coverage, and paving, which limit opportunities for active mobility and physical activity in daily life.

Beyond the residential area, sociodemographic characteristics, such as educational attainment and skin color, were also consistently associated with the quality of the microenvironment for engaging in leisure physical activity and as a means of commuting. In the present study, individuals with higher educational attainment and those of White or Asian skin color had higher scores for an environment conducive to physical activity. Similar findings were observed in the city of Los Angeles, United States^26^. Studies have identified significant disparities among minority populations based on criteria related to income, education, race, and mobility, with racial minorities such as African Americans and Hispanics being exposed to unfavorable urban conditions.

Access routes to parks in areas occupied by these groups had heavier vehicle traffic and poorer walking conditions. Neighborhoods with a high presence of minorities also exhibited greater urban deterioration and fewer positive aesthetic features. Just as observed in high-income countries, in upper-middle-income countries such as Brazil, education level and skin color also constitute central social markers of environmental inequities. National evidence reinforces this scenario: data from the Instituto Brasileiro de Geografia e Estatística (IBGE - Brazilian Institute of Geography and Statistics) indicate that the Black and Brown populations face persistent, historically constructed disadvantages in areas such as income, education, housing conditions, and access to goods and services, factors directly related to the quality of the urban environment and opportunities for adopting active lifestyles^27^.

For example, sidewalks, which are essential features that influence pedestrians’ experiences, encourage urban mobility, especially for those who rely on public transportation or walk. In line with the findings of this study, a 2019 survey conducted by the Center for Metropolitan Studies showed that people from lower socioeconomic classes and Black individuals lived in areas with poorer sidewalk conditions. It is worth noting that the *Pesquisa Origem e Destino* (Origin and Destination Survey)^28^, conducted by the São Paulo Metro, surveyed people from 32,000 households in 39 municipalities in the São Paulo metropolitan region and showed that 46% of trips in Greater São Paulo are made on foot, highlighting the need to ensure adequate infrastructure for these trips.

Regarding skin color, it was observed that areas with a higher concentration of Black and Brown people have higher mixed-use land-use scores, possibly due to historical patterns of urban occupation and the need for access to nearby basic services. Furthermore, racial segregation and structural inequalities may have shaped these territories, resulting in greater diversity of businesses, services, and residences. Adaptation to these socioeconomic and historical conditions led these communities to develop a built environment that facilitates mobility and access to essential resources^29^.

Therefore, discussing disparities from the perspective of educational attainment and skin color involves recognizing that these characteristics intersect^5^. Skin color and educational attainment can predict and explain complex scenarios of inequality, in which individuals from racial minority groups and those with lower incomes frequently face cumulative barriers in accessing opportunities and resources, including the quality of the urban environment^27^.

Regarding different age groups, older adults had the highest scores for sidewalks, aesthetics, and tree coverage. It is believed that this result may also be related to socioeconomic factors. From a social perspective, it may be that older people, especially those with greater financial stability and higher education, are living in better-structured areas^30^.

Environmental interventions, based on policies such as the 2014 Strategic Master Plan, had the primary objective of reducing environmental inequities in São Paulo. However, based on this study, it is emphasized that the microenvironment for engaging in leisure physical activity and as a means of commuting remains unequal in São Paulo. New policies, actions, and programs should prioritize areas with lower socioeconomic status to increase access to better environments and, thereby, offer greater opportunities for people to be physically active. Educational initiatives should also be developed to raise public awareness that the environments in which they live are critical determinants of health and behavior.

This is the first study on the microenvironment for leisure physical activity and commuting in São Paulo, one of the ten most populous cities in the world. A key strength was the inclusion of both sides of the streets, broadening the characteristics assessed. Another strength is the use of micro-scale environmental measures in different regions of a megalopolis with many inequalities, such as São Paulo, where analyses of different regions can help shape public policies aimed at improving the environment. Furthermore, as this is an innovative study, this data will allow for international comparisons.

Among the limitations, the sample, although distributed throughout the city, does not represent all residents. The MAPS Global method may not fully reflect participants’ experiences, as the analyzed route (425m–725m to a shopping center) may differ from actual trips. Reliance on Google Street View imagery, especially in outlying areas with infrequent updates, may compromise the accuracy of the analysis.

## CONCLUSION

The micro-scale environment surrounding the residences of people living in central areas of the city of São Paulo had higher scores. People with higher levels of education, White or Asian skin color, and aged 60 years or older had higher microenvironment scores in the surroundings of their residences.

The findings underscore the need to develop urban public policies and initiatives in areas such as the eastern and southern regions of São Paulo, and for people in situations of greater social vulnerability, such as black and brown individuals and those with low levels of education, who generally live in areas of the city with lower socioeconomic status. These actions can contribute to spatial justice and provide greater opportunities for people to choose and adopt healthy behaviors, such as engaging in leisure physical activity and as a means commuting.

## Data Availability

The dataset supporting the results of this study is not publicly available.
