# Supplementary material for: Differences in the microenvironment for leisure physical activity and commuting in São Paulo
Source: Rev Saude Publica. 2026 Jun 12;60:e21. doi: 10.11606/s1518-8787.2026060006996 (PMC13262925; doi:10.11606/s1518-8787.2026060006996)
Supplement: Translation

# Translation: Diferenças no microambiente para atividade física no lazer e deslocamento em São Paulo

**Authors:** Elaynne Silva de Oliveira, Inaian Pignatti Teixeira, Ítalo Vinícius Floriano de Paula, Jader Correia de Lacerda, Rildo de Souza Wanderley, Alexandre Augusto de Paula da Silva, Adriano Akira Ferreira Hino, Alex Antonio Florindo

**Affiliations:** I Universidade Federal do Maranhão. Pinheiro, MA, Brasil; II Universidade do Estado de Minas Gerais. Passos, MG, Brasil; III Universidade de São Paulo. Faculdade de Medicina. São Paulo, SP, Brasil; IV Universidade de São Paulo. Escola de Artes, Ciências e Humanidades. São Paulo, SP, Brasil; V Universidade de Pernambuco. Escola Superior de Educação Física. Recife, PE, Brasil; VI Washington University in St Louis. School of Public Health. Prevention Research Center. St. Louis, MO, United States; VII Pontifícia Universidade Católica do Paraná. Escola de Ciências da Vida. Programa de Pós-Graduação em Ciências da Saúde. Curitiba, PR, Brasil

**Keywords:** Ambiente Construído, Atividade Física, Iniquidades

## Abstract

**OBJETIVO:**

Descrever as características do ambiente construído em microescala e verificar diferenças conforme a região de moradia e variáveis sociais e demográficas entre adultos em São Paulo, Brasil.

**MÉTODOS:**

Trata-se de uma análise transversal com 1.434 adultos da coorte ISA–Atividade Física e Ambiente. As características do ambiente foram avaliadas pelo método *Microscale Audit of Pedestrian Streetscapes* (MAPS Global) a partir de imagens do Google Earth em rotas de 400m–700m dos endereços georreferenciados dos participantes. Para análise, foram aplicados testes U de Mann-Whitney para dois grupos independentes e Kruskal-Wallis para três ou mais grupos, seguido do pós-teste de Dunn, para verificar diferenças nos escores médios do ambiente segundo coordenadorias de saúde, sexo, idade, escolaridade e cor da pele.

**RESULTADOS:**

O entorno das residências na região Centro-Oeste apresentou os maiores escores gerais (p < 0,001). Indivíduos com maior escolaridade, autodeclarados brancos ou amarelos e com 60 anos ou mais residiam em áreas com melhores escores de estética, aspectos sociais, presença de árvores, coberturas aéreas, calçadas, cruzamentos e escore geral do ambiente (p < 0,001). Em contrapartida, pessoas com menor escolaridade e autodeclaradas pretas ou pardas viviam em áreas com piores condições de calçadas (p < 0,001).

**CONCLUSÃO:**

Conclui-se que as condições de microescala foram melhores no entorno das residências em regiões centrais e para indivíduos com maior escolaridade, pele branca ou amarela e mais velhos, ressaltando a necessidade de políticas públicas para um planejamento urbano mais equitativo e inclusivo.

## INTRODUÇÃO

O ambiente urbano e o planejamento das cidades influenciam a saúde da população, afetando fatores como sedentarismo, baixos níveis de atividade física, poluição do ar e sonora, violência e isolamento social^1^. Em países com menor disponibilidade histórica de infraestrutura urbana adequada, essas relações tendem a ser mais evidentes, especialmente diante do rápido crescimento populacional nos centros urbanos e de processos de urbanização marcados por planejamento insuficiente ao longo de décadas^2^. No contexto brasileiro, país classificado como de renda média-alta segundo o Banco Mundial, estima-se que, em 2023, cerca de 85% da população vivia em áreas urbanas^3^, e projeta-se que, até 2050, aproximadamente 70% da população mundial residirá nessas áreas⁴. Essa rápida urbanização configura-se como um desafio global, uma vez que as cidades se tornam cenários centrais na produção de iniquidades em saúde⁵, reforçando a importância do planejamento urbano orientado à promoção de cidades mais saudáveis.

Estudos sobre atividade física destacam disparidades em saúde, com evidências de que sexo^6^, renda^7^, escolaridade^8^ e raça/etnia^9^ influenciam estas práticas, principalmente no lazer e como forma de deslocamento. Embora fatores individuais sejam relevantes, a interação com o ambiente é crucial. A vizinhança, moradia e bairros impactam a saúde^10^, ressaltando a importância do planejamento urbano para a saúde pública. Evidências de países de alta renda como Estados Unidos, Inglaterra e Austrália, de acordo com a classificação do Banco Mundial^11^, indicam que o espaço urbano tende a ser desigual para grupos mais vulneráveis^12^. Apesar das diferenças socioeconômicas e culturais, No Brasil, estudo em Curitiba mostrou menor presença de árvores e elementos estéticos nas periferias, refletindo desigualdades semelhantes^13^.

Espaços públicos acessíveis, calçadas de qualidade, conectividade viária e segurança no trânsito são essenciais para promover atividades físicas, especialmente em países de baixa e média renda^14^. São Paulo foi marcada por um planejamento urbano desordenado, impulsionado pela industrialização acelerada e por processos migratórios e imigratórios desordenados. Por exemplo, entre 1950 e os anos 2000, a população subiu de dois milhões para mais de dez milhões de habitantes^15^. A falta de planejamento provocou problemas como desigualdade espacial, acesso a transportes públicos, mobilidade urbana, saneamento básico, impermeabilização do solo, poluição do ar e sonora, enchentes, iniquidades nas áreas verdes, déficit habitacional e habitações precárias.

No entanto, a partir de 2010, foram implementadas políticas importantes para a redução das iniquidades ambientais^16^. Entre 2015 e 2020, houve aumento na infraestrutura para atividade física, como academias ao ar livre, praças, parques e ciclovias, impulsionado principalmente pelo Novo Plano Diretor e pelo Plano de Mobilidade Urbana. Porém, essas melhorias ainda são mais comuns em áreas de maior nível socioeconômico. Por exemplo, Teixeira et al.^17^ demonstraram que, apesar do crescimento de estruturas que favorecem a prática de atividades físicas, como as ciclovias, ainda há predominância em áreas de maior nível socioeconômico na cidade. Variáveis do microambiente ou em microescala, como presença e qualidade de calçadas, controladores de tráfego, como semáforos, lombadas, aspectos estéticos e coberturas verdes, como a presença de árvores, são muito importantes para a promoção da atividade física.

No entanto, a literatura ainda apresenta uma lacuna significativa no que diz respeito a investigações sobre o microambiente para a atividade física, especialmente em países de baixa e média renda como o Brasil, e em cidades marcadas por profundas desigualdades, como São Paulo. A capital paulista enfrenta problemas complexos como alta densidade populacional, tráfego desordenado e desigualdade de renda, que podem impactar na saúde pública. Portanto, faz-se importante investigar o microambiente para as práticas de atividades físicas no lazer e como deslocamento de forma aprofundada em cidades como São Paulo, Brasil, que representam bem as características de megalópolis do Sul Global, onde ainda existem muitas desigualdades sociais e ambientais. Este estudo poderá subsidiar políticas públicas de habitação, infraestrutura urbana, mobilidade urbana, vigilância em saúde e promoção de ambientes saudáveis. Diante do exposto, o presente estudo tem como objetivo descrever as características da microescala do ambiente para prática de atividade física e verificar possíveis diferenças segundo regiões de moradia, níveis de escolaridade e cor da pele de adultos da cidade de São Paulo, Brasil.

## MÉTODOS

### Cenário da Pesquisa

São Paulo é a cidade mais populosa do Brasil e da América Latina, com uma população estimada de 11.451.999 milhões de habitantes, com área de 1.521,110 km^2^ e densidade populacional de 7.528,26 habitantes por km^17^. A cidade é dividida em 96 distritos, 31 subprefeituras, seis áreas de saúde e no ano de 2022 tinha 27.301 setores censitários. São Paulo concentra cerca de 11% do produto interno bruto do país e tem índice Gini de 0,539, que a coloca como uma das cidades com maior desigualdade de renda, que pode refletir no acesso à saúde, à habitação e ao transporte^3,18^.

### Tipo de Estudo

Este estudo é do tipo transversal e utiliza dados da coorte “Inquérito de Saúde de São Paulo (ISA): Atividade Física e Ambiente”, que tem como objetivo primário investigar se o ambiente construído está associado práticas de atividades físicas no lazer e como forma de deslocamento, além de objetivos secundários como o estudo do comportamento sedentário, obesidade, doenças cardiovasculares e doenças mentais entre adultos residentes da cidade de São Paulo. Maiores detalhes do ISA–Atividade Física e Ambiente, podem ser obtidos no estudo de Florindo et al.^19^

### Amostra

A amostra deste estudo incluiu 1.434 pessoas que foram entrevistadas na segunda onda (2020/2021), que são residentes nas seis áreas administrativas de saúde (Leste, Sudeste, Centro-Oeste, Norte e Sul) na cidade de São Paulo. Maiores detalhes sobre a amostragem do ISA-Atividade Física e Ambiente podem ser obtidos em artigo publicado por Florindo et al.^20^ que descreve o perfil da coorte.

### Questionários

O estudo ISA–Atividade Física e Ambiente utilizou questionário estruturado para ser aplicado via entrevistas telefônicas na segunda onda (2020/2021), devido à pandemia de covid-19. A coleta ocorreu utilizando a metodologia *Computer Assisted Telephone Interviewing*, similar à empregada no Sistema de Vigilância de Fatores de Risco e Proteção para Doenças Crônicas por Inquérito Telefônico do Ministério da Saúde.

O questionário da segunda onda consistiu em 70 questões, organizadas em 11 blocos temáticos: identificação das pessoas (seis questões), atividade física (30 questões), mobilidade (quatro questões), avaliação do sono (três questões), peso e altura (duas questões), percepção de saúde (uma questão), doenças (oito questões), tabagismo (uma questão), comportamento na pandemia (três questões), características socioeconômicas (duas questões) e características da família e do domicílio (dez questões).

### Avaliação do Microambiente para a Prática de Atividades Físicas

O microambiente para a prática de atividade física, também compreendido como o nível de avaliação em microescala, refere-se ao ambiente de pequena escala ao redor das pessoas, cujas condições e características podem influenciar de forma significativa a decisão de praticar atividade física no tempo de lazer ou como forma de deslocamento^10^. Em geral, o microambiente envolve avaliações de ruas, segmentos e cruzamentos e verifica a presença e qualidade de calçadas, presença e qualidade de espaços como ciclovias, parques e praças, segurança no trânsito, avaliando a presença de controladores de tráfego de veículos, aspectos estéticos como paisagismo e pichações, presente de estruturas de iluminação, cobertura vegetal, a presença e o tamanho de prédios e a presença de serviços comerciais, instituições de ensino e religiosas, instalações públicas e privadas.

A avaliação do microambiente realizada por meio do *Microscale Audit of Pedestrian Streetscapes-Global* (MAPS*-Global*)^21^. Foram considerados 1.434 adultos avaliados na segunda onda do estudo (2020/2021), cujos 1.205 endereços residenciais foram georreferenciados para a construção das rotas. A auditagem ocorreu online, com imagens do Google Earth, em rotas de 400 e 725 metros, distância que corresponde, aproximadamente, ao deslocamento realizado por uma pessoa em até dez minutos de caminhada. Esse intervalo está de acordo com o padrão adotado pelo manual do instrumento MAPS Global. O *Google Street View* foi utilizado para percorrê-las. A partir dos endereços, identificou-se o centro comercial como o setor censitário com maior concentração de registros no Cadastro Nacional de Pessoa Jurídica (CNPJ), obtidos via Imposto Predial e Territorial Urbano de São Paulo. Os 794.968 registros de CNPJ foram georreferenciados, e cada setor censitário recebeu uma marcação espacial indicando seu centro comercial.

Para aumentar a cobertura da avaliação, optou-se por analisar os dois lados das ruas (direito e esquerdo). A avaliação do ambiente ocorreu entre dezembro de 2020 e setembro de 2022. Ao todo, foram analisadas 1.205 rotas, 11.276 segmentos e 11.222 cruzamentos, com um tempo médio de 25,8 minutos por rota (dp = 17,4 minutos). As imagens utilizadas no *Google Street View* dataram de janeiro de 2010 (mais antiga) a maio de 2022 (mais recente).

A auditoria foi realizada por uma equipe composta por sete avaliadores previamente treinados, dos quais três também atuaram como supervisores durante o processo de coleta de dados. Os supervisores receberam treinamento específico ministrado pelo grupo coordenador do MAPS no Brasil e, posteriormente, foram responsáveis por capacitar os demais avaliadores envolvidos no estudo. Como parte do processo de padronização e controle de qualidade, todos os auditores passaram por testes de confiabilidade interavaliadores, conforme preconizado pelo método MAPS. Apenas os avaliadores que atingiram o nível mínimo de concordância de 90% exigido pelo instrumento participaram da coleta de dados. Todos os processos metodológicos estão descritos detalhadamente no estudo de Oliveira et al.^22^

Os itens avaliados pelo MAPS *Global* utilizam diferentes formatos de resposta (dicotômicas, categóricas e contínuas), exceto as questões de uso misto do solo, que variam de 0 a 5. Para calcular os escores, as respostas dicotômicas (não/sim) são pontuadas como 0 ou 1. Itens individuais e subescalas são pontuados, gerando subescalas de valências positivas (associadas com mais chances de realizar atividades físicas) e negativas (associadas com menos chances de realizar atividades físicas). As escalas e subescalas geram os escores de cada seção (rotas, segmentos e cruzamentos). Para análises e cálculos, seguiu–se a padronização e sistematização de pontuações adotadas pela metodologia do instrumento.

### Variáveis de Estudo

#### Variáveis do microambiente

Para gerar os escores do ambiente construído foram consideradas três seções do instrumento: rotas, segmentos e cruzamentos, totalizando 148 itens organizados em escalas e escores principais do MAPS *Global.* A seção de rotas analisa a diversidade de infraestrutura urbana, incluindo uso do solo residencial e comercial, disponibilidade de serviços, mobilidade e qualidade estética do ambiente. A seção de segmentos examina detalhadamente os trechos entre cruzamentos, considerando infraestrutura para pedestres e ciclistas, características das calçadas, presença de sombras, vendedores ambulantes e elementos que impactam a segurança e a experiência do pedestre. Já a seção de cruzamentos avalia a acessibilidade e segurança para travessias, verificando sinalização, largura das vias, semáforos, rampas e faixas de pedestres.

Para a análise, foram consideradas as escalas: uso misto do solo, uso das ruas, estética e ambiente social, infraestrutura para pedestres, árvores e coberturas aéreas, calçadas, características negativas das calçadas, facilidades para pedestres e acessibilidade. Também foram analisados os escores principais, rotas, segmentos, cruzamentos e o escore geral do instrumento. Os segmentos e cruzamentos foram apresentados como médias por rota, evitando discrepâncias individuais. Inicialmente, as variáveis desses elementos foram somadas e, depois, calculadas as médias dos atributos, antes da soma das pontuações gerais de cada subescala, escala e escore geral. Na análise das rotas, não foi necessário calcular médias, pois cada indivíduo possuía apenas uma rota.

## Variáveis Demográficas, Sociais e Regiões de Residência

Foram incluídos dados de pessoas com 18 anos ou mais (n = 1.434, residentes em 1.205 domicílios) avaliadas até a segunda onda do estudo (2020/2021). As variáveis consideradas foram: sexo (homens e mulheres) e cor da pele (brancos e amarelos; pretos, pardos e indígenas), ambas coletadas na linha de base e utilizadas como padrão em toda a coorte; escolaridade (nível 1: até o 5° ano do ensino fundamental; nível 2: até a 2° série do ensino médio; nível 3: ensino médio completo; e nível 4: ensino superior incompleto ou mais), e idade (18 a 39 anos; 40 a 59 anos; e 60 anos ou mais), ambas coletadas na segunda onda.

Foram utilizadas as regiões de moradia dos participantes classificadas pelas Coordenadorias de Saúde que estavam vigentes na linha de base do estudo em 2014/2015 (Norte, Sul, Leste, Sudeste e Centro-Oeste), devido à seleção da amostra e para possíveis comparações do microambiente no entorno dos domicílios onde as pessoas viviam.

## Análises dos Dados

Foram realizados testes de Kolmogorov-Smirnov para identificar se as variáveis possuíam aderência à distribuição normal, mostrando que todas as variáveis do ambiente construído tinham distribuição não paramétrica. Para as análises descritivas, foram calculadas as medianas, os intervalos interquartis, os valores mínimos e máximos, além do percentual de ausência dos atributos agregados em escalas e nas seções de rotas, segmentos e cruzamentos.

As diferenças entre os escores médios do ambiente construído, foram analisadas com base nas diferentes regiões de moradia, níveis de escolaridade, cor da pele e grupos etários. Em seguida, foram realizados testes U de Mann-Whitney para dois grupos independentes e teste de Kruskal-Wallis para três ou mais grupos, seguido do pós-teste de Dunn para identificar os grupos significativos estatisticamente.

Todos os dados foram analisados utilizando o SPSS versão 24.0 (SPSS, Inc., Chicago, IL). Para todos os testes, considerou-se significância estatística para p < 0,05.

## Questões Éticas

O estudo foi aprovado pelos Comitês de Ética da Escola de Artes, Ciências e Humanidades da Universidade de São Paulo (10396919.0.0000.5390) e da Secretaria Municipal de Saúde de São Paulo (10396919.0.3001).

## RESULTADOS

A maioria da amostra era do sexo feminino, com idade até 59 anos, com escolaridade com ensino médio ou superior, se autodeclararam de cor de pele branca ou amarela e residiam nas coordenadorias de saúde sul e sudeste (Tabela 1).

**Tabela 1:** Características sociais e demográficas da amostra em 2020/2021, estudo ISA-Atividade Física e Ambiente, São Paulo, SP, Brasil.

Variável sociodemográfica	n	%
Sexo		
Feminino	840	58,6
Masculino	594	41,4
Faixas etárias		
18 a 39 anos	489	34,1
40 a 59 anos	432	30,1
≥ 60 anos	513	35,8
Escolaridade^a^		
Até o 5° ano do ensino fundamental	251	17,5
Até a 2° série do ensino médio	284	19,8
Ensino médio completo	470	32,8
Ensino superior incompleto ou mais	419	29,2
Cor da pele^a^		
Brancos e amarelos	768	53,6
Pretos, pardos e indígenas	656	45,7
Coordenadorias de saúde de moradia		
Centro-Oeste	201	14,0
Leste	259	18,1
Norte	279	19,5
Sudeste	326	22,7
Sul	369	25,7
Total	1.434	100

ISA: Inquérito de Saúde de São Paulo.

^a^ Dez pessoas com valores *missing*.

Verificou–se que atributos como uso misto do solo, árvores e coberturas aéreas e calçadas estão presentes na maioria das rotas. No entanto, elementos como infraestrutura e acessibilidade para pedestres são características ausentes em metade ou mais das rotas (Tabela 2).

**Tabela 2:** Escalas e escores gerais das rotas avaliadas do MAPS Global de acordo com o endereço residencial dos participantes (n = 1.434 de 1.205 endereços avaliados), estudo ISA-Atividade Física e Ambiente, São Paulo, SP, Brasil.

Escala e escore MAPS	Itens/pontos	Mediana	Intervalo interquartil	Mínimo–Máximo	% ausência do atributo
Escala					
Uso misto do solo	38 (0 a 162)	17,0	12 a 21	0 a 43	0,1
Uso das ruas	20 (0 a 24)	5,0	2,7 a 7,0	0 a 15	5,3
Estética e ambiente social	5 (0 a 11)	2,0	1,0 a 4,0	-3 a 9	19,7
Infraestrutura para pedestres	3 (0 a 3)	0,1	0 a 0,3	0 a 1	88,0
Arvores e coberturas aéreas	3 (0 a 21)	3,5	2,6 a 4,6	0 a 10	0,9
Calçadas	2 (0 a 12)	9,2	8,2 a 10,3	0 a 12	0,1
Características negativas em calçadas	6 (0 a 36)	10,5	8,8 a 12,7	0 a 20	0,1
Facilidades para pedestres	7 (0 a 7)	0,5	0,2 a 0,7	0 a 3	49,3
Acessibilidade	3 (0 a 6)	0,0	0 a 0,5	0 a 6	69,7
Escore					
Rotas		24,0	18 a 30	0 a 57	
Segmentos		10,8	8,5 a 14,2	-4 a 39	
Cruzamentos		1,0	0,3 a 2,0	-1 a 13	
Escore geral do instrumento		37,1	28,7 a 45,9	1 a 105	

ISA: Inquérito de Saúde de São Paulo; MAPS: *Microscale Audit of Pedestrian Streetscapes.*

O entorno das residências das pessoas que viviam na região Centro-Oeste, obteve os maiores escores nas escalas de estética, árvores e coberturas aéreas, acessibilidade, rotas, segmentos, cruzamentos e escore geral de ambiente quando comparado ao entorno das outras regiões. A região Centro-Oeste apresentou também o menor escore de características negativas das calçadas. Em contrapartida, a região Sul apresentou menores valores para estética e facilidade para pedestres (Tabela 3).

**Tabela 3:** Escalas e escores gerais do microambiente avaliadas no entorno das residências dos indivíduos (n = 1.434 de 1205 endereços) de acordo as regiões de residências estabelecidas pelas coordenadorias de saúde em 2015, estudo ISA-Atividade Física e Ambiente, São Paulo, SP, Brasil.

	Escala e escore MAPS				
	Centro-Oeste (1)	Leste (2)	Norte (3)	Sudeste (4)	Sul (5)
**Mediana (intervalo interquartil)**					
Escala					
Uso misto do solo	16,0 (9,0–24,0)	18,0 (14,0–21,0)	17,0 (12,0–21,0)	14,0 (10,0–18,25)^a^	18,0 (12,0–23,0)
Uso das ruas	5,0 (3,0–7,0)	5,0 (2,0–7,0)	5,0 (2,75–7,0)	5,0 (2,0–7,0)	5,0 (2,0–7,0)
Estética e ambiente social	3,0 (2,0–5,0)^a^	2,0 (1,0–3,0)	2,0 (1,0–4,0)	3,0 (2,0–4,0)	1,0 (0–3,0)^a^
Infraestrutura para pedestres	0,1 (0–0,3)^a^	0 (0–0,2)	0,1 (0–0,3)	0 (0–0,2)^a^	0,1 (0–0,3)
Árvores e coberturas aéreas	4,7 (3,3–6,7)^a^	3,25 (2,4–4,2)	3,5 (2,6–4,6)	3,6 (2,9–4,6)	3,1 (2,5–4,0)
Calçadas	9,3 (8,1–10,4)	9,7 (8,4–10,7)	9,2 (8,2–10,3)	9,1 (8,1–10,0)	8,8 (8,0–9,8)^a^
Características negativas das calçadas	9,8 (8,4–11,1)^a^	11,2 (8,7–13,0)	10,5 (8,4–13,0)	10,8 (8,5–12,0)	11,1 (9,7–13,1)
Facilidades para pedestres	0,5 (0,2–1,0)	0,4 (0,2–0,7)	0,5 (0,2–0,7)	0,5 (0,25–0,78)	0,3 (0–0,6)^a^
Acessibilidade	0,2 (0,0–1,9)^a^	0 (0–0,5)	0 (0–0,5)	0 (0–0,5)	0 (0–0,5)
Escore					
Rotas	24,0 (15,0–34,0)^a^	23,0 (19,0–31,0)	24,00 (18,0–30,0)	22,0 (17,0–26,0)	24,0 (19,0–31,0)
Segmentos	16,4 (10,5–21,8)^a^	10,5 (8,8–13,4)	10,8 (8,5–10,8)	11,5 (7,2–14,7)	9,4 (6,2–11,2)
Cruzamentos	1,3 (0,5–3,7)^a^	0,7 (0,2–1,8)	1,0 (1,0–2,0)	1,1 (0,5–2,0)	0,8 (0–1,5)
Escore geral do instrumento	43,0 (32,5–57,3)^a^	36,0 (28,6–46,5)	37,1 (28,7–45,9)	35,42 (27,2–44,8)	34,5 (26,7–43,3)

ISA: Inquérito de Saúde de São Paulo; MAPS: *Microscale Audit of Pedestrian Streetscapes.*

Nota: p < 0,05 = teste de Kruskal Wallis.

^a^ Valor estatisticamente significativo e diferente das demais medianas.

Quando se comparou o entorno das residências de acordo com os níveis de escolaridade das pessoas (Tabela 4), indivíduos com nível superior possuíam valores superiores nas escalas positivas de estética e ambiente social, árvores e coberturas aéreas, facilidade para pedestres, acessibilidade, escores de rotas, segmentos, cruzamentos e escore geral de ambiente, além de menores escores de características negativas das calçadas.

**Tabela 4:** Escalas e escores gerais do microambiente no entorno das residências de acordo com os níveis de escolaridade da amostra (n = 1.424 de 1.205 endereços), estudo ISA–Atividade Física e Ambiente, São Paulo, SP, Brasil.

Escala e escore MAPS	Escolaridade			
	Nível 1	Nível 2	Nível 3	Nível 4
**Medianas (intervalo interquartil)**				
Escala				
Uso misto do solo	17,0 (11,0–21,0)	17,0 (12,0–21,0)	17,0 (12,0–21,0)	16,0 (11,0–21,0)
Uso das ruas	5,0 (2,0–7,0)	5,0 (2,0–7,0)	5,0 (2,0–7,0)	5,0 (3,0–8,0)
Estética e ambiente social	2,0 (1,0–3,0)	2,0 (1,0–3,0)	2,0 (1,0–4,0)	3,0 (1,0–4,0)^a^
Infraestrutura para pedestres	0,1 (0–0,3)	0,1 (0–0,3)	0,1 (0–0,2)	0,1 (0–0,3)
Árvores e coberturas aéreas	3,3 (2,5–4,4)	3,2 (2,4–4,2)	3,4 (2,6–4,5)	3,8 (3,0–5,4)^a^
Calçadas	9,2 (8,1–10,4)	9,1 (8,0–10,1)	9,3 (8,2–10,3)	9,3 (8,3–10,3)
Características negativas das calçadas	11,1 (9,1–13)	10,5 (9,0–12,7)	10,6 (9,0–13,0)	10,2 (8,6–12,0)^a^
Facilidades para pedestres	0,4 (0,2–0,7)	0,4 (0,1–0,6)	0,5 (0,2–0,8)	0,5 (0,2–0,9)^a^
Acessibilidade	0 (0–0,5)	0 (0–0,5)	0 (0–0,5)	0 (0–0,9)^a^
Escore				
Rotas	24,0 (17,0–31,0)	24,0 (19,0–30,0)	23,0 (18,0–29,0)	24,0 (18,0–30,0)
Segmentos	10,1 (7,8–13,2)	10,5 (8,5–13,4)	10,6 (8,2–13,9)	12,1 (9,0–17,7)^a^
Cruzamentos	1,0 (0,3–1,7)	0,8 (0,2–1,6)	1,0 (0,4–2,0)	1,1 (0,4–2,7)^a^
Escore geral do instrumento	35,8 (26,8–45,7)	35,7 (28,4–45,5)	36,6 (28,6–44,7)	39,2 (31,3–49,0)^a^

ISA: Inquerito de Saude de Sao Paulo; MAPS: *Microscale Audit of Pedestrian Streetscapes*.

Nota: p < 0,05 = teste de Kruskal Wallis.

^a^ Valor estatisticamente significativo e diferente das demais medianas.

Verificou–se que pessoas com 60 anos ou mais apresentaram os maiores valores nas escalas positivas de estética, presença de árvores e coberturas aéreas, bem como o escore de segmentos. Além disso, esse grupo teve menores escores em características negativas das calçadas (Tabela 5).

**Tabela 5:** Escalas e escores gerais do microambiente de acordo com as faixas de idade e cor da pele (n = 1.434 de 1.205 endereços), estudo ISA-Atividade Física e Ambiente, São Paulo, SP, Brasil.

Escala e escore MAPS	Faixa etária (idade)		
	18 a 39 anos	40 a 59 anos	≥ 60 anos
**Medianas (intervalo interquartil)**			
Escala			
Uso misto do solo	17,0 (12,0–21,0)	16,0 (12,0–21,0)	17,0 (11,0–21,0)
Uso das ruas	5,0 (2,0–7,0)	5,0 (3,0–7,0)	5,0(3,0–8,0)
Estéticas e características sociais	2,0 (1,0–4,0)	2,0 (1,0–4,0)	**3,0 (1,0–4,0)** ^a^
Infraestrutura para pedestres	0,1 (0–0,3)	0,1(0–0,3)	0,1 (0–0,2)
Árvores e coberturas aéreas	3,5 (2,7–4,4)	3,3 (2,6–4,5)	**3,6 (2,7–4,8)** ^a^
Calçadas	9,3 (8,2–10,3)	9,2 (8,1–10,2)	9,2 (8,2–10,3)
Características negativas de calçadas	10,6 (9,0–12,8)	10,6 (9,0–13)	**10,2 (8,7–12,3)** ^a^
Facilidades para pedestres	0,5 (0,1–0,7)	0,4 (0,2–0,7)	0,5 (0,2–0,7)
Acessibilidade	0 (0–0,5)	0 (0–0,5)	0,1 (0–0,6)
Escore			
Rotas	24,0 (18–30,0)	23,0 (18–29)	24,0 (17–30)
Segmentos	10,5 (8,0–13,7)	10,6 (8,5–13,8)	**11,5 (8,6–15,3)** ^a^
Cruzamentos	0,9 (0,3–2,0)	0,9 (0,3–2,0)	1,1 (0,4–2,0)
Escore geral do instrumento	37,0 (28,5–45,6)	36,4 (28,6–45,7)	37,7 (28,7–46,9)
**Escala e escore MAPS**	**Cor da pele**		
	**Brancos e amarelos**	**Pretos e pardos**	
**Medianas (intervalo interquartil)**			
Escala			
Uso misto do solo	18,0 (12,0–24,0)	**19,0 (14–24,0)**	
Uso das ruas	5,0 (2,0–8,0)	5,0 (2,0–7,0)	
Estéticas e características sociais	**2,0 (1,0–4,0)** ^a^	2,0 (0–3,0)	
Infraestrutura para pedestres	**0,1 (0–0,3)**	0,1 (0–0,2)	
Arvores e coberturas aéreas	**3,6 (2,8–4,7)** ^a^	3,2 (2,5–4,2)	
Calçadas	**9,4 (8,3–10,3)**	9,1 (8,0–10,1)	
Características negativas das calçadas	10,2 (8,6–12,4)	**10,7 (9,1–13,0)** ^a^	
Facilidades para pedestres	**0,5 (0,2–0,8)**	0,4 (0,1–0,7)	
Acessibilidade	**0,1 (0–0,7)**	0 (0–0,5)	
Escore			
Rotas	24,0 (18,0–30,0)	24,0 (17,7,0–29,0)	
Segmentos	**11,5 (8,8–15,1)** ^a^	10,0 (7,9–13,6)	
Cruzamentos	**1,1 (0,4–2,3)** ^a^	0,8 (0,2–1,7)	
Escore geral do instrumento	**38,8 (29,7–47,0)** ^a^	35,4 (27,2–43,8)	

ISA: Inquérito de Saúde de São Paulo; MAPS: *Microscale Audit of Pedestrian Streetscapes.*

Nota: p < 0,05 = teste de Kruskal Wallis para idade e Teste de Mann–Whitney U para cor de pele.

^a^ Valor estatisticamente significativo; as medianas em negrito são estatisticamente diferentes das medianas sem negrito, indicando diferenças significativas entre os grupos comparados.

Em relação à cor da pele, indivíduos autodeclarados brancos e amarelos apresentaram maiores escores de infraestrutura para pedestres, acessibilidade, estética, presença de árvores e coberturas aéreas, segmentos, cruzamentos e no escore geral do ambiente. Por outro lado, os maiores escores de uso misto do solo e de características negativas das calçadas, foram observados entre indivíduos autodeclarados pretos e pardos (Tabela 5).

## DISCUSSÃO

Este estudo teve como objetivo descrever as características do microambiente construído entre adultos e idosos participantes da coorte ISA-Atividade Física e Ambiente e verificar diferenças segundo as regiões de residência e características demográficas e sociais. Pessoas que residiam em regiões centrais, de maior escolaridade e de cor de pele branca ou amarela tiveram maiores escores de ambientes para as práticas de atividades físicas de lazer e como deslocamento.

Quanto aos resultados por região de residência, sabe-se que, em São Paulo, SP, as áreas centrais concentram pessoas de maior nível socioeconômico. Além disso, estudos demonstram que estruturas que favorecem a prática de atividades físicas no lazer e no deslocamento ativo, como estações de trem e metrô, ciclovias e praças, estão mais presentes nessas regiões centrais da cidade⁸. Esses achados evidenciam a existência de desigualdades territoriais na distribuição de atributos do ambiente construído associados à prática de atividade física.

Embora ainda sejam escassos, os estudos que avaliam o microambiente para a prática de atividade física no Brasil e em países da América Latina, especialmente aqueles que utilizam instrumentos padronizados de auditoria ambiental, como o MAPS, as evidências disponíveis apontam para um padrão consistente de desigualdade espacial. Estudo desenvolvido em Curitiba^13^, Brasil, utilizando o MAPS, reforça esse cenário ao avaliar o microambiente no entorno de 30 escolas públicas, demonstrou que a qualidade do ambiente construído diminui à medida que se afasta do centro urbano, com escores mais elevados nas áreas centrais e piores condições nas regiões periféricas. Em um segundo momento, o estudo também evidenciou que essas áreas periféricas, majoritariamente ocupadas por grupos de menor renda, apresentam menor acesso a serviços urbanos e a elementos estéticos e de conforto, como arborização e arte urbana.

Já um estudo realizado no município de Cambé^23^, no estado do Paraná, que utilizou análises de clusters, para examinar os agrupamentos formados a partir da relação entre a caminhabilidade em microescala, a renda por setor censitário e os níveis de caminhada, estes últimos obtidos a partir do Plano de Mobilidade de Cambé (PR). Evidenciou que as regiões de maior renda e aquelas com maior movimentação de pedestres apresentaram as maiores médias de pontuação geral de caminhabilidade em seus respectivos trechos. Esses achados reforçam a existência de uma associação positiva entre melhores condições do ambiente construído em microescala e a prática da caminhada, ao mesmo tempo em que revelam desigualdades socioespaciais na distribuição de infraestruturas urbanas favoráveis à mobilidade ativa.

As discrepâncias na qualidade do ambiente urbano no Brasil resultam de fatores históricos, legais e da desigualdade social, refletindo processos de urbanização desordenada e ocupação irregular, como observado em São Paulo, onde persistem grandes contrastes de infraestrutura e condições de vida^24^. Dados do Censo Demográfico 2022^25^ reforçam que essas desigualdades estão fortemente associadas aos locais de moradia, evidenciando que moradores de favelas e comunidades urbanas convivem com infraestrutura significativamente mais precária, especialmente em atributos centrais da microescala pedestre, como calçadas, acessibilidade, arborização e pavimentação, o que limita as oportunidades de mobilidade ativa e prática de atividade física no cotidiano.

Para além da região de residência, características sociodemográficas, como escolaridade e cor da pele, também se associaram de forma consistente à qualidade do microambiente para a prática de atividades físicas no lazer e como forma de deslocamento. No presente estudo, pessoas com maior escolaridade e de cor da pele branca ou amarela apresentaram maiores escores de ambiente favorável à atividade física. Achados semelhantes foram observados na cidade de Los Angeles, Estados Unidos^26^. Estudos identificaram iniquidades significativas em populações minoritárias, a partir de critérios relacionados à renda, escolaridade, raça e mobilidade, nas quais minorias raciais consideradas como afro-americanos e hispânicos eram expostos a condições urbanas desfavoráveis.

As rotas de acesso a parques em áreas ocupadas por esses grupos apresentavam maior tráfego de veículos e piores condições de caminhada. Bairros com alta presença de minorias, também exibiam maior deterioração urbana e menos características estéticas positivas. Assim como observado em países de alta renda, em países de renda média-alta, como o Brasil, a escolaridade e a cor da pele também configuram marcadores sociais centrais das iniquidades ambientais. Evidências nacionais reforçam esse cenário: dados do Instituto Brasileiro de Geografia e Estatística indicam que a população preta e parda apresenta desvantagens persistentes e historicamente construídas em dimensões como renda, educação, condições de moradia e acesso a bens e serviços, fatores diretamente relacionados à qualidade do ambiente urbano e às oportunidades de adoção de comportamentos ativos^27^.

Por exemplo, no caso das calçadas, que são atributos essenciais que influenciam as experiências dos pedestres, estimulam o deslocamento urbano, especialmente para quem depende do transporte público ou se desloca a pé. Em linha com os achados do presente estudo, um levantamento feito em 2019 pelo Centro de Estudos da Metrópole demonstrou que pessoas de classes sociais mais baixas e de cor da pele preta viviam em áreas com piores condições de calçadas. Ressalta-se que a Pesquisa Origem e Destino^28^, realizada pelo Metrô de São Paulo, ouviu pessoas de 32 mil domicílios em 39 municípios da região metropolitana de São Paulo e mostrou que 46% das viagens na Grande São Paulo são realizadas a pé, evidenciando a necessidade de garantir uma infraestrutura adequada para esses deslocamentos.

Ainda quanto à cor da pele, observou-se que áreas com maior presença de pessoas pretas e pardas apresentam maiores escores de uso misto do solo, possivelmente devido a padrões históricos de ocupação urbana e à necessidade de acesso a serviços básicos próximos. Além disso, a segregação racial e as desigualdades estruturais podem ter moldado esses territórios, resultando em maior diversidade de comércios, serviços e residências. A adaptação a essas condições socioeconômicas e históricas levou essas comunidades a desenvolverem um ambiente construído que facilita a mobilidade e o acesso a recursos essenciais^29^.

Portanto, discutir disparidades a partir da ótica da escolaridade e cor da pele, envolve reconhecer que essas características se cruzam de maneira interseccional^5^. Cor da pele e escolaridade podem predizem e explicar cenários complexos de desigualdade, em que indivíduos de grupos raciais minoritários e com menor renda frequentemente enfrentam barreiras cumulativas no acesso a oportunidades e recursos, incluindo a qualidade do ambiente urbano^27^.

Com relação às diferentes faixas etárias, as pessoas idosas apresentaram os maiores escores em calçadas, estética e cobertura de árvores. Acredita-se que este resultado também possa estar relacionado a fatores socioeconômicos. Do ponto de vista social, pode ser que pessoas mais velhas, especialmente aquelas com maior estabilidade financeira e escolaridade, estejam vivendo em regiões mais estruturadas^30^ .

Intervenções ambientais, a partir de políticas como o Plano Diretor Estratégico de 2014, tiveram como principal objetivo reduzir as iniquidades ambientais em São Paulo. No entanto, a partir deste estudo, ressalta-se que o microambiente para as práticas de atividades físicas no lazer e como forma de deslocamento ainda permanece desigual em São Paulo. Novas políticas, ações e programas devem priorizar locais de menor nível socioeconômico para que se possa aumentar o acesso a ambientes melhores e, assim sendo, oferecer maiores oportunidades de escolha para as pessoas serem ativas fisicamente. Também se devem desenvolver ações educacionais para que a população esteja ciente de que os ambientes onde elas vivem são determinantes para a saúde e para os comportamentos.

Este é o primeiro estudo sobre microambiente para atividades físicas no lazer e deslocamento em São Paulo, uma das dez cidades mais populosas do mundo. Um ponto forte foi a inclusão de ambos os lados das ruas, ampliando as características avaliadas. Outro ponto forte é o uso de medidas ambientais em microescala, em diferentes regiões de uma megalópole com muitas iniquidades como São Paulo, onde as análises de diferentes regiões pode fomentar políticas públicas de melhora do ambiente. Além disso, por ser um estudo inovador, estes dados vão permitir comparações internacionais.

Entre as limitações, a amostra, embora distribuída por toda a cidade, não representa todos os residentes. O método MAPS Global pode não refletir plenamente as experiências dos participantes, pois a rota analisada (425m–725m até um centro comercial) pode diferir dos deslocamentos reais. A dependência de imagens do *Google Street View*, especialmente em áreas periféricas com atualizações esparsas, pode comprometer a precisão da análise.

## CONCLUSÃO

O ambiente em microescala no entorno das residências de pessoas que viviam em regiões centrais da cidade de São Paulo apresentou escores mais elevados. Pessoas com maior escolaridade, de cor de pele branca ou amarela e com 60 anos ou mais, tiveram maiores escores de microambiente no entorno das suas residências.

Os achados reforçam a necessidade de formulação de políticas públicas urbanas e ações em áreas como as regiões Leste e Sul de São Paulo, e para pessoas em contexto de maior vulnerabilidade social, como os pretos e pardos e de baixa escolaridade, que em geral vivem em regiões de menor nível socioeconômico na cidade. Estas ações podem contribuir para a justiça espacial e para maior oportunidade de as pessoas escolherem e adotarem comportamentos saudáveis como as práticas de atividades físicas no lazer e como forma de deslocamento.

## Data Availability

O conjunto de dados que dá suporte aos resultados deste estudo não está disponível publicamente.
